# Connecting gut microbiome composition to symptom severity and host genetics in endometriosis

**DOI:** 10.3389/fmicb.2026.1873766

**Published:** 2026-10-05

**Authors:** Giuseppe Giovanni Nardone, Lara Emily Rosso, Aurora Santin, Beatrice Spedicati, Stefania Cappellani, Maria Pina Concas, Gabriella Zito, Federico Romano, Giovanni Di Lorenzo, Nunzia Zanotta, Giuseppina Campisciano, Giuseppe Ricci, Manola Comar, Giorgia Girotto

**Affiliations:** 1 Institute for Maternal and Child Health, IRCCS “Burlo Garofolo”, Trieste, Italy; 2 Department of Medicine, Surgery and Health Sciences, University of Trieste, Trieste, Italy

**Keywords:** endometriosis, gut microbiome, host–microbiome interaction, inflammation, symptoms, taste-associated genes

## Abstract

**Background:**

Endometriosis (EM) is a multifactorial, estrogen-dependent inflammatory disease characterized by high clinical variability. Growing evidence is highlighting the role of gut microbiome dysbiosis in EM pathophysiology; however, associations between microbiome, symptom severity, and host genetics remain poorly characterized. Extra-oral taste receptor genes (TGs), expressed in both the gastrointestinal and female reproductive systems, may represent novel mediators of gut-reproductive tract axis crosstalk.

**Methods:**

A total of 50 women with surgically confirmed EM were deeply clinically characterized, including the evaluation of EM-symptoms and their severity. Microbiome profiles were obtained by 16S rRNA gene sequencing of rectal swabs. Genetic variants within four TGs (*TAS2R3, TAS2R4, TAS2R5,* and *TAS2R14*) were assessed using SNP-array genotyping. Microbiome composition was investigated through the evaluation of alpha and beta diversity, as well as enterotype classification analyses. Associations between EM-related features, bacterial taxa and TG-SNPs were investigated using regression models.

**Results:**

Three distinct gut microbiome community types were identified, dominated by *Bacteroides*, *Prevotella*, and *Escherichia–Shigella*. While microbiome composition did not differ significantly across EM stages, beta diversity was associated with dysmenorrhea (R^2^ = 0.13) and disease stage (R^2^ = 0.12). Regarding TGs, SNPs within *TAS2R14* gene showed significant associations with alpha and beta diversity indices. Reduced abundance of short-chain fatty acid–producing taxa, including members of the *Lachnospiraceae* and *Ruminococcaceae* families, was associated with dysmenorrhea, whereas increased abundance of *Prevotella* and *Mobiluncus* with dysuria and infertility, respectively. Finally, an increased premenstrual pain severity was associated with a reduced relative abundance of taxa within the order *Burkholderiales* (*β* = −2.265) and the family *Sutterellaceae* (*β* = −1.563); high postmenstrual pain severity was associated with a lower abundance of *Bifidobacteriales* (*β* = −4.932) and *Bifidobacteriaceae* (*β* = −4.134).

**Conclusion:**

This case-only study elucidated the complex relationship between gut microbiome composition, EM-related symptoms, and host genetic variation. In particular, extra-oral taste receptor genes emerged as novel risk factors to host–microbiome interactions, supporting their potential role in the microbiota-endometriosis axis and opening new perspectives on EM pathogenesis.

## 1 Introduction

Endometriosis (EM) is an estrogen-driven, inflammatory, gynecological disorder affecting 10–15% of women of reproductive age (Moradi et al., 2021; Shen et al., 2025). It is characterized by the ectopic proliferation of endometrial tissue outside the uterus, from organs located in the pelvis (e.g., ovaries, fallopian tubes, bladder, and bowel) to tissues in the abdominal and thoracic cavities (e.g., lungs) (Zondervan et al., 2018; Nezhat et al., 2019; Yao et al., 2023). EM is associated with a broad spectrum of symptoms, including dysmenorrhea, dyspareunia, and dyschezia, all of which can impair daily activities, social interactions, and overall quality of life (Zondervan et al., 2018, 2020). In many cases, these symptoms may persist chronically, contributing to fatigue, psychological distress, and even reduced work productivity. Significantly, fertility issues are also present; indeed, infertility affects up to 50% of women with EM, further enhancing the burden of the disease (Carvalho et al., 2013; Da Broi et al., 2019a).

Currently, EM is recognized as a multifactorial condition, meaning that its onset and progression are driven by genetic predisposition and environmental factors, with a prominent role of the immune system, hormone metabolism, and microbiome. From a genetic standpoint, more than 40 susceptibility loci have been associated with EM risk in Genome-Wide Association Studies (Guare et al., 2025); in parallel, increasing attention is being directed toward the contribution of rare variants to disease development (Santin et al., 2023), facilitated by the expanding application of next-generation sequencing technologies in the study of this condition.

In this framework, a growing number of studies are highlighting a possible role of extra-oral taste-receptor genes (TGs) in complex disorders etiopathogenesis. Indeed, beyond influencing our food choices (Di Pizio et al., 2019), they are expressed in multiple body districts, including the gut, in which they are reported to operate as nutrient and chemical sensors within endocrine and immune networks (Behrens et al., 2014) and interact with the microbiota (Feng et al., 2018a). However, no studies to date have investigated TGs in the context of EM.

Regarding the microbiome, recent evidence suggests that the gut microbiota might play a pivotal role in the pathophysiology of EM potentially contributing to both disease development and symptomatology (Guo and Zhang, 2024).

In physiological condition, the human intestinal flora is mainly composed of *Firmicutes* and *Bacteroidota* phyla which account together for 90% of the total microbial community (Qin et al., 2010). *Firmicutes* are Gram-positive bacteria comprising mainly *Bacillus, Clostridium, Enterococcus, Lactobacillus,* and *Ruminococcus* (Rinninella et al., 2019) while *Bacteroidotas* are Gram-negative and include around 7.000 species, primarily from *Bacteroides, Alistipes, Parabacteroides,* and *Prevotella* (Gibiino et al., 2018). The *ratio* between these two dominant phyla (the *Firmicutes/Bacteroidota* (F/B) ratio) has been standardly used to define microbial homeostasis. An increase in the F/B ratio is generally linked to dysbiosis (Abenavoli et al., 2019); nevertheless, other minor phyla may contribute to this condition without altering this ratio (Shin et al., 2015).

Several studies describe an association between EM and dysbiosis as perturbations in the composition of the gut microbiota have been consistently reported in affected individuals. In particular, F/B ratio imbalance observed in EM patients has been linked to estrogen metabolism dysregulation thereby potentially promoting disease progression (Gloux et al., 2011; Flores et al., 2012; Kovács et al., 2021). Under this framework, intestinal microbiota plays a crucial role in promoting EM progression by increasing the reabsorption of free estrogen through the secretion of *β*-glucuronidase and *β*-glucosidase enzymes (Saponara et al., 2025).

Indeed, according to the literature, gut dysbiosis could act as a driver of EM pathogenesis via the ‘bacterial contamination hypothesis’, that states that shifts in the gut microbiota composition can compromise intestinal epithelial tight junctions, thus increasing mucosal permeability and causing the systemic leakage of lipopolysaccharides, into the peritoneal cavity (Khan et al., 2018). This triggers the inflammatory cascade by activating macrophages via the TLR4 signaling pathway, compromising immunosurveillance mechanisms and fostering an aberrant production of pro-inflammatory cytokines; this allows the establishment of a sustained inflammatory environment that plays a pivotal role in the initiation and progression of EM (Tai et al., 2018).

Notably, also studies on animal models show that EM induces significant gut microbiota shifts compared to healthy controls (Yuan et al., 2018). Furthermore, causal links have been explored through fecal microbiota transplantation from EM mice into recipient mice promotes lesion formation and inflammation (Chadchan et al., 2023).

Moreover, alterations in the gut microenvironment may exert systemic consequences, including effects on the central nervous system that may lead to altered central sensitization of the mechanisms underlying chronic pain. Indeed, metabolites produced by the gut microbiota, including gamma-aminobutyric acid, and short-chain fatty acids (SCFA), are capable of interacting with neuronal receptors, thereby modulating pain-related signaling pathways (Salliss et al., 2021). Nevertheless, research specifically addressing EM-associated symptoms remains limited.

Beyond pain modulation, the gut microbiome is also increasingly recognized as a key regulator of female fertility. Gut dysbiosis has been linked to ovarian dysfunction and impaired ovulatory processes, whereas the restoration of microbial balance via prebiotics and probiotics has demonstrated beneficial effects on reproductive outcomes, including improved pregnancy rates (Qi et al., 2021). These findings suggest that targeting the gut microbiota may hold therapeutic potential not only for the general management of EM but also for patients specifically affected by EM-related infertility.

To this regard, recent studies are highlighting also the potential involvement of TGs in female fertility and gut–reproductive system communication. Specifically, bitter taste receptors (*TAS2Rs*), traditionally recognized for detecting bitter compounds, have been shown to be expressed in somatic ovarian follicle cells, namely granulosa and cumulus cells, with *TAS2R14* identified as the most abundantly expressed subtype Importantly, altered expression of these receptors has been linked to fertility issues, as disruptions in ovarian TAS2R-mediated signaling may impair follicular development, oocyte competence, and implantation potential, all particularly relevant mechanisms in EM (Semplici et al., 2021). Based on this evidence demonstrating the specific expression and functional relevance of *TAS2R3, TAS2R4, TAS2R5,* and *TAS2R14* in human follicular tissue, these four receptors could represent key primary candidates for investigating host-genetic influences along the gut–reproductive tract axis.

Given the growing recognition of the gut microbiota-endometriosis *axis axis*, in which gut microbial communities modulate estrogen metabolism, immune tolerance, and inflammatory pathways relevant to reproductive health, it is plausible that TAS2Rs could serve as molecular mediators bridging gut-derived signals and reproductive system function (Pérez-Prieto et al., 2024). Consequently, deepening their role could provide novel mechanistic insights underlying EM development and EM-related infertility; nevertheless, no studies are available in the literature.

To this end, we aimed to investigate the complex interplay among EM clinical features, microbiome composition, and genetic variants within TGs, with the goal of identifying novel mechanisms underlying disease severity, and modulating EM symptomatology,

The aims and overall workflow of the study are illustrated in Figure 1.

**Figure 1:**
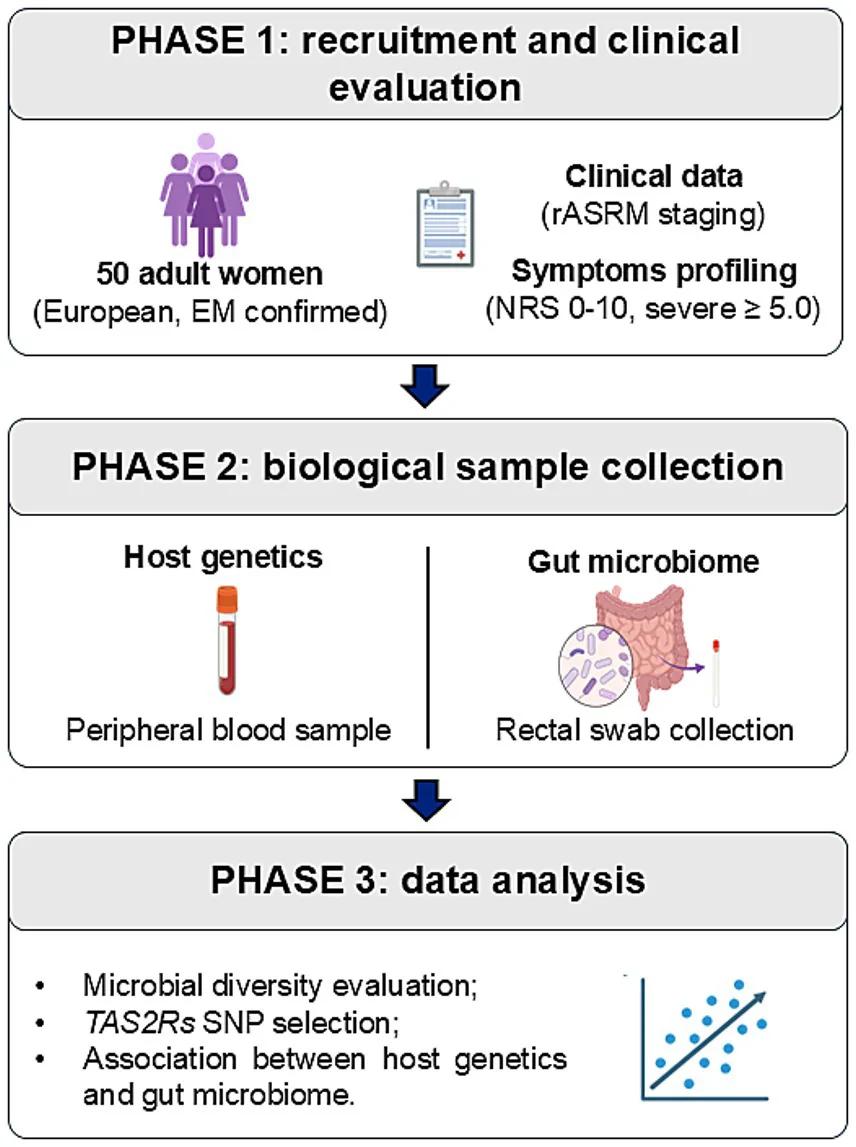
Study workflow overview. Schematic representation of this study design. Phase 1: recruitment of 50 adult women with confirmed EM, clinical data collection (rASRM staging), and symptom profiling. Phase 2: biological sampling, including peripheral blood for genetic analyses and rectal swabs for assessment of the gut microbiome. Phase 3: genetic analysis of the selected extra-oral *TAS2R* genes, microbiome profiling, integration of genetic and microbiome data.

## 2 Materials and methods

### 2.1 Ethical statement

Written informed consent to participate in the study and for the collection of biological samples for research purposes was obtained from all patients. The study was conducted in accordance with the Helsinki Declaration and was approved by the Ethics Committee of the Friuli-Venezia Giulia region (Italy) (Prot. n. 47,846 dd. 22.12.2022).

### 2.2 Participants’ recruitment and data collection

In this case-only study, a total of 50 women with hystologically confirmed surgical diagnosis of EM were recruited at the I.R.C.C.S. “Burlo Garofolo” Hospital in Trieste (Italy). These patients are routinely evaluated at our Institute as part of our standard annual clinical and surgical activities for EM. The following inclusion criteria were employed: (i) age over 18 years old, (ii) a laparoscopically and histologically confirmed surgical diagnosis of EM, (iii) European genetic ancestry, to minimize host population stratification in genetic analyses. Exclusion criteria included presence of active infectious diseases at the time of enrolment.

Disease stage was determined according to the revised American Society for Reproductive Medicine (rASRM) classification (Canis et al., 1997). All participants underwent a comprehensive clinical assessment; anamnestic and gynecological data, including ongoing medical therapies and infertility diagnosis, were collected through dedicated clinical reports.

As previously described (Santin et al., 2023), an evaluation of the most common EM-associated symptoms (e.g., ovulation pain, premenstrual pain, postmenstrual pain, chronic pelvic pain, dysmenorrhea, dyspareunia, dyschezia, and dysuria) was performed. For each symptom, presence was recorded as a dichotomous variable (“yes”/“no”), while severity was rated on a 0–10 Numerical Rating Scale (NRS). Furthermore, in order to classify patients according to EM-symptom severity, a dichotomous variable was created. For each symptom, a score of 1 was assigned when the reported intensity on the Numerical Rating Scale (NRS) was ≥5.0 (classified as “Intense pain”), whereas a score of 0 was attributed to cases with an intensity <5.0 (classified as “Mild pain”) according to (Boonstra et al., 2016).

All data were stored in the Research Electronic Data Capture (REDCap) platform, a secure, web-based application developed by Vanderbilt University for the collection, management, and reuse of research data (https://www.project-redcap.org).

Finally, peripheral blood samples were collected from all patients for genetic analyses, and rectal swabs were obtained for microbiome profiling.

### 2.3 Genetic analyses: SNP-array genotyping

Genomic DNA was extracted from peripheral blood samples of EM patients using the QIAsymphony® SP instrument with the QIAsymphony® Midi Kit (Qiagen, Venlo, The Netherlands). DNA quality was assessed by 1% agarose gel electrophoresis, and concentration was determined using a NanoDrop ND-1000 spectrophotometer (NanoDrop Technologies Inc., Wilmington, DE, USA). SNP-array genotyping was conducted with the following platforms: (i) Illumina GSA-24v3-0-EA chip (ii) Illumina GSAMD-24v3-0-EA chip (Illumina Inc., San Diego, CA, USA).

Standard quality control (QC) was conducted applying the following parameters: sample call-rate ≥0.95. Hardy–Weinberg Equilibrium *p*-value >1 × 10^−6^, and minor allele frequency (MAF) ≥ 0.01. Imputation was conducted on the TOPMed Imputation Server (Taliun et al., 2019).

### 2.4 Genetic analyses: TGs-variants selection

A candidate gene approach was employed to select target loci. Rather than profiling the entire human taste receptor family, we restricted our analysis to four specific genes (*TAS2R3, TAS2R4, TAS2R5*, and *TAS2R14*) based on peer-reviewed evidence confirming their localized expression and signaling roles within human ovarian follicle cells, involved in female fertility regulation (Semplici et al., 2021),and expression in final intestinal tract according to the Adult Genotype Tissue Expression database (GTeX, https://www.gtexportal.org/home/, accessed on April 10th 2026). SNPs were extracted employing *bcftools* software (version 1.14) plug-in split-vep (https://samtools.github.io/bcftools/howtos/plugin.split-vep.html) with an INFO score ≥0.6.

The selection of TG-SNPs included in the study focused on tagging (tSNP) and their functional role in the female reproductive system, as described in (Farinella et al., 2021). As a first step, the choice of the tSNPs was based on Linkage Disequilibrium (LD) in the European population. Specifically, the Haploview software (https://www.broadinstitute.org/haploview/haploview version 4.2) was employed to choose tSNPs with the following criteria: r^2^ > 0.8 and MAF > 0.05. Only SNPs described to have a role in regulating gene expression in the female reproductive tract, namely expression Quantitative Trait Loci (eQTLs) were selected. To this purpose, the LINkage Disequilibrium-based Annotation (LinDA) browser (http://linda.irgb.cnr.it/index.html) was employed. The final list consisted of 12 TG-SNPs (Supplementary Table 1).

### 2.5 Microbiome analyses: DNA extraction and library preparation

To evaluate gut microbiome composition, rectal swabs were collected shortly before the surgical procedure; although stool samples are traditionally used, rectal swabbing was chosen because previous studies demonstrated that rectal swabs are highly representative of the overall luminal gut microbial diversity (Budding et al., 2014; Turner et al., 2022; Marin et al., 2025). Specifically, rectal swabs (APTACA Spa, Regione Monforte, Canelli, Italy) were inserted 1–3 cm to ensure adequate sampling of the distal rectum, followed by storage at −80 °C in 1.5 mL of sterile saline solution.

Following thawing, samples were vortexed, and total DNA was extracted from 300 μL of each sample, with a final elution volume of 50 μL, utilizing the Maxwell CSC DNA Blood Kit (Promega, Madison, WI, USA) in accordance with the manufacturer’s protocol.

The bacterial composition of samples was analyzed by sequencing the V3 region of the 16S rRNA gene. A qPCR targeting the V1-V3 region (500 bp) of the 16S rRNA gene was conducted using U534R and the degenerated primer 27FYM. A subsequent semi-nested PCR was performed with primers B338F_P1-adaptor and U534R_A_adaptor_barcode, targeting the V3 region (200 bp) of the 16S rRNA gene, each sample being barcoded by the reverse primer (Sundquist et al., 2007). PCR reactions were executed with EvaGreen® dye (Fisher Molecular Biology, Waltham, MA, USA), Kapa 2G HiFi Hotstart ready mix 2X (Kapa Biosystems, Wilmington, MA, USA), 0.5 μM of each primer, and 400 ng/μL of Bovine Serum Albumin (BSA), in a 10 μL final volume. Temperature cycling conditions included: an initial 5 min at 95 °C, 30 s at 95 °C, 30 s at 59°/57 °C, 45 s at 72 °C, and a 10 min final elongation step at 72 °C. Negative controls were included, processed concurrently with clinical samples from the pre-analytic phase. Amplicon sizes were validated as 560 bp for the initial PCR and 260 bp for the semi-nested PCR on a 2% agarose gel. Post-semi-nested PCR, the dsDNA concentration in each sample was measured using a Qubit® 2.0 Fluorimeter (Invitrogen, Carlsbad, California, USA) with the Qubit® dsDNA BR Assay Kit (Thermo Fisher Scientific, Waltham, MA, USA). Equimolar amounts of each sample (100 ng) were pooled to construct a library at 100 pM final concentration according to the manufacturer’s guidelines. Template preparation was facilitated by emulsion PCR using the Ion OneTouch™ 2 System (Life Technologies, Gran Island, New York, NY, USA), utilizing the Ion PGM Hi-Q View OT2 200 kit (Life Technologies, New York, NY, USA). Subsequent quality control was performed using a Qubit® 2.0 Fluorimeter. Sequencing was conducted with the Ion PGM™ System technology using the Ion PGM Hi-Q View sequencing kit (Life Technologies, New York, NY, USA).

### 2.6 Microbiome analyses: quality control and taxonomic classification

The quality of FastQ files was initially assessed utilizing FastQC v0.11.8 (https://www.bioinformatics.babraham.ac.uk/projects/fastqc/) and MultiQC v1.0 (Ewels et al., 2016) to detect outliers based on read quality metrics. Subsequently, Trimmomatic v0.36 (Bolger et al., 2014) was employed to filter out low-quality reads by eliminating sequences that failed to achieve an average quality score of at least 25 within a 4-nucleotide sliding window. Additionally, reads shorter than 50 base pairs were discarded. Negative controls did not pass this quality filtering step and contained no analyzable sequences, indicating that contamination was negligible. Following trimming, read quality was re-assessed using FastQC and MultiQC. Information on input, surviving and dropped reads during the Qc process is available in Supplementary Table 11.

The bacterial composition of the samples was characterized utilizing the DADA2 package v1.34 (Callahan et al., 2016). Subsequent to the removal of sequences shorter than 160 bp and the elimination of chimeras, taxonomic classification was executed using the Silva database v138 (Quast et al., 2013). Rare amplicon sequence variants (ASVs) resulting from DADA2 were removed; only ASVs that had at least 5 reads in at least 2 samples were maintained for subsequent analyses.

### 2.7 Microbiome analyses: diversity evaluation

Alpha diversity indices, including species richness, the Shannon index, and the Simpson index, were calculated using the breakaway package v4.8.4 (Willis and Martin, 2020) as well as the DivNet package v0.4.0 (Willis and Martin, 2020). Disparities in Alpha diversity indices among samples were examined via the “*betta()*” function (Willis et al., 2017) within the breakaway package. Using Canberra distances, variations in Beta diversity among samples were evaluated through Principal Coordinates Analysis (PCoA) and assessed using permutational analysis of variance (PERMANOVA) through the *adonis2()* function from the vegan v2.7 package. Each permanova was executed specifying the “by = onedf” parameter, to assess the impact of each covariate included in the model on Beta diversity. Beta dispersion for groups with significant results from PERMANOVA was evaluated using the “*betadisper()*” and the “*permutest()*” functions from the vegan v2.7 package. Both PERMANOVA and breakaway alpha diversity models were corrected using the following covariates: age, Body Mass Index (BMI), current hormonal therapy, total read count per sample. All statistical tests employed the Bonferroni correction for multiple comparisons. A *p*-value of less than 0.05 was established as the threshold for statistical significance.

### 2.8 Microbiome analyses: community typing

Enteric community types were determined utilizing a Dirichlet multinomial mixtures (DMM) model, selecting the cluster number with the lowest Laplace approximation. Differences in alpha and beta diversity among enteric clusters were then evaluated using the “betta_lincom()” function from the breakaway package for alpha diversity and pairwise PERMANOVA for beta diversity. All statistical analyses were corrected using the Bonferroni correction setting 0.05 as the significance threshold.

### 2.9 Microbiome analyses: linear regression models

Linear models were employed to examine the relationships between bacterial taxa and selected phenotypic traits. Specifically, we analyzed bacterial abundance in relation to: (1) EM-associated conditions and symptoms (i.e., $EM_{\text{related phenotypes}},$ including EM stage, infertility status, dysmenorrhea, ovulation pain, premenstrual pain, postmenstrual pain, pelvic pain, dyspareunia, dyschezia, and dysuria); (2) severity of EM-related symptoms; and (3) genotypes of the previously selected TG-SNPs having a functional role in the female reproductive system. To address the compositional nature of the data, taxa relative abundance was normalized employing the Centered Log Ratio (CLR) transformation. Furthermore, taxa were subjected to a filtering process for a prevalence of 10% among the samples. The models were organized as showed in Equations (1–3):

$$\mathrm{Tax}a_{\mathrm{CLR}-\text{transformed}}∼EM_{\text{related phenotypes}}+\text{Covariates}$$ (1)

$$EM_{\text{symptoms intensity}}∼\mathrm{Tax}a_{\mathrm{CLR}-\text{transformed}}+\text{Covariates}$$ (2)

$$\mathrm{Tax}a_{\mathrm{CLR}-\text{transformed}}∼\text{Tast}e_{\text{related}\;\mathrm{SNP}\;\text{genotype}}+\text{Covariates}$$ (3)

*Taxa_CLR-transformed_* refers to bacterial abundances transformed using the CLR transformation. The placeholder “Covariates” refers to the covariates used to correct all models: age, Body Mass Index (BMI), current hormonal therapy, total read count per sample. Analyses were carried out for all taxonomic levels (*Phylum*, *Class*, *Order*, *Family*, and *Genus*). In all models models, an additive genetic inheritance model was assumed, with genotypes coded as 0, 1, or 2 copies of the effect allele. For each taxonomical rank, analyses were corrected using the Bonferroni correction, setting 0.05 as the significance threshold.

## 3 Results

### 3.1 Cohort description and symptoms evaluation

A cohort of 50 EM patients aged between 25 and 55 years old (mean age of 37.82 ± 8.6 years) was enrolled at I. R. C. C. S. “Burlo Garofolo” Hospital (Trieste, Italy). Overall, 37 patients (74%) were diagnosed with the most severe forms of EM (i.e., stage III-IV), while milder diagnosis of EM stage I-II was detected in 13 (26%) patients. Of the total, 29 patients (58%) were receiving ongoing medical therapy. Complete demographic and clinical data are reported in Table 1.

**Table 1:** Demographic and clinical data of EM patients.

Demographic and clinical data *N* = 50	Mean ± sd or *N* (%)
Age	
Mean (SD)	37.82 ± 8.76
Range	25.00, 55.00
BMI	
Mean (SD)	23.00 ± 4.25
Range	16.63, 35.64
Diagnosis (rASRM classification)	
EM stage I	4 (8.0%)
EM stage II	9 (18.0%)
EM stage III	12 (24.0%)
EM stage IV	25 (50.0%)
Ongoing medical therapy	29 (58.0%)
EM-infertility diagnosis	7 (14.0%)
Pregnancies	
Yes	30 (60.0%)
No	20 (40.0%)

The table reports the main demographic and clinical data of EM patients described as mean, standard deviation (Mean ± sd) and range or number (*N*) and percentage (%) of patients. EM diagnosis staged according to the revised American Society for Reproductive Medicine classification (rASRM). Ongoing medical therapy: number (*N*) and percentage (%) of patients currently undergoing a medical therapy for EM. EM-infertility diagnosis: number (*N*) and percentage (%) of patients diagnosed with EM-related infertility.

At enrolment, patients underwent a comprehensive assessment of EM-related symptoms. The most frequently reported were dysmenorrhea (66%), ovulation pain (47%) and dyspareunia (46%) (Table 2). Notably, the mean severity for all reported EM symptoms was rated above 5.00 on a 0-10-point scale, indicating a high intensity of symptom severity.

**Table 2:** EM symptoms presence and severity assessment.

Symptoms evaluation and intensity assessment	*N* (%)
Dysmenorrhea (presence)	33 (66%)
Severity (NRS)	
Mean (SD)	6.82 ± 1.83
Range	3.00, 10.00
Ovulation pain (presence)	23 (47%)
Severity (NRS)	
Mean (SD)	6.14 ± 2.18
Range	2.00, 10.00
Premenstrual pain (presence)	20 (40%)
Severity (NRS)	
Mean (SD)	5.97 ± 2.25
Range	2.00, 9.00
Postmenstrual pain (presence)	11 (22%)
Severity (NRS)	
Mean (SD)	7.41 ± 2.11
Range	3.5, 10.00
Pelvic pain (presence)	15 (31%)
Severity (NRS)	
Mean (SD)	5.86 ± 2.48
Range	1.00, 9.00
Dyspareunia (presence)	23 (46%)
Severity (NRS)	
Mean (SD)	5.88 ± 2.39
Range	2.00, 10.00
Dyschezia (presence)	16 (32%)
Severity (NRS)	
Mean (SD)	5.50 ± 1.71
Range	2.00, 8.00
Dysuria (presence)	5 (10%)
Severity (NRS)	
Mean (SD)	5.75 ± 2.49
Range	2.00, 8.00

The table reports the EM-symptoms evaluation, assessed in terms of presence of the specific EM-related symptom and its relative severity, rated on the Numerical Rating Scale (NRS), before medical therapy or surgery, or at baseline. Mean and standard deviation (Mean, SD) for continuous variables are reported, while categorical ones with number (*N*) and percentage (%) of patients. For each parameter, percentages were calculated on the number of patients with available data.

A distinctive feature of EM is the lack of correlation between anatomical disease stage and the clinical burden of pain symptoms. Patients with advanced-stage EM may exhibit minimal or no symptoms, while those with lower-stage disease can experience severe, disabling, and therapeutically refractory pain. Consistent with this heterogeneity, our analysis revealed no statistically significant associations between the rASRM stage and the presence or severity of pain-related symptoms (Supplementary Table 2 and Supplementary Figure 1).

### 3.2 Taxonomic composition of gut microbiome in early and late stages of EM

The taxonomic composition at the genus and phylum levels of the gut microbiome was evaluated in patients with early (I-II) and late (III-IV) stages of EM (Figure 2).

**Figure 2:**
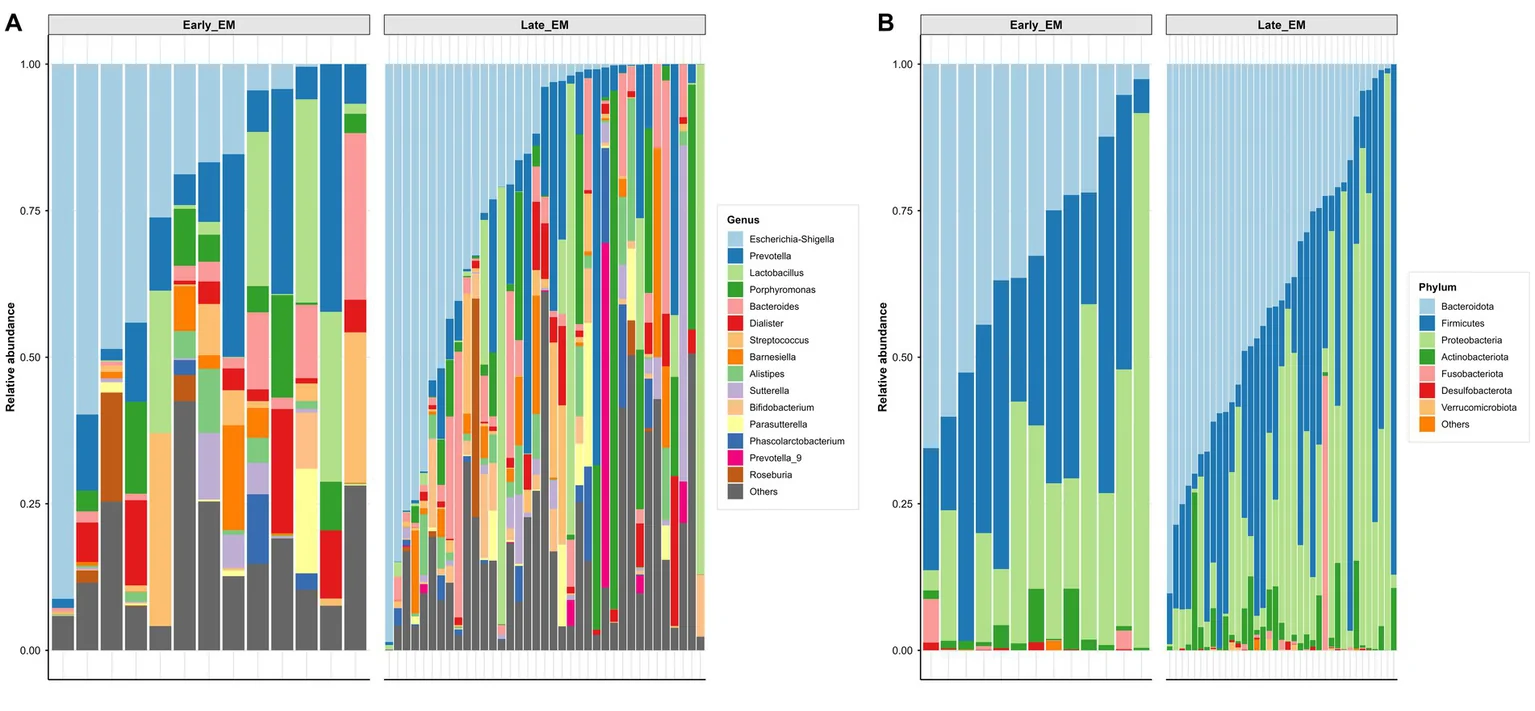
Graphic representing relative abundance of gut microbiota in patients with early (Early_EM) and late stages (Late_EM) of EM. **(A)** Taxonomic composition at genus level. The most prevalent genera reported are *Escherichia-Shigella* (in light blue), *Prevotella* (in blue) and *Lactobacillus* (in light green). **(B)** Taxonomic composition at phylum level. The most prevalent phylum reported is *Bacteroidota* (in light blue), followed by *Firmicutes* (in blue) and *Proteobacteria* (in light green).

In both groups, the most prevalent genus was *Escherichia-Shigella* followed by *Prevotella* and *Lactobacillus*. Regarding the phyla, the most prevalent ones in order of abundance were *Bacteroidota*, *Firmicutes* and *Proteobacteria*, in line with previous studies reporting that *Escherichia-Shigella* genera and *Bacteroidota* phylum are increased in EM (Quaranta et al., 2019; Qin et al., 2022; Baușic et al., 2025).

### 3.3 Community composition analysis

Gut microbiome community types were determined utilizing a DMM model, which allowed to classify patients into three distinct enterotypes: C1, predominantly enriched in *Bacteroides*; C2, characterized by a dominance of *Prevotella*; and C3, notably distinguished by a high abundance of *Escherichia–Shigella* (Figure 3B).

**Figure 3:**
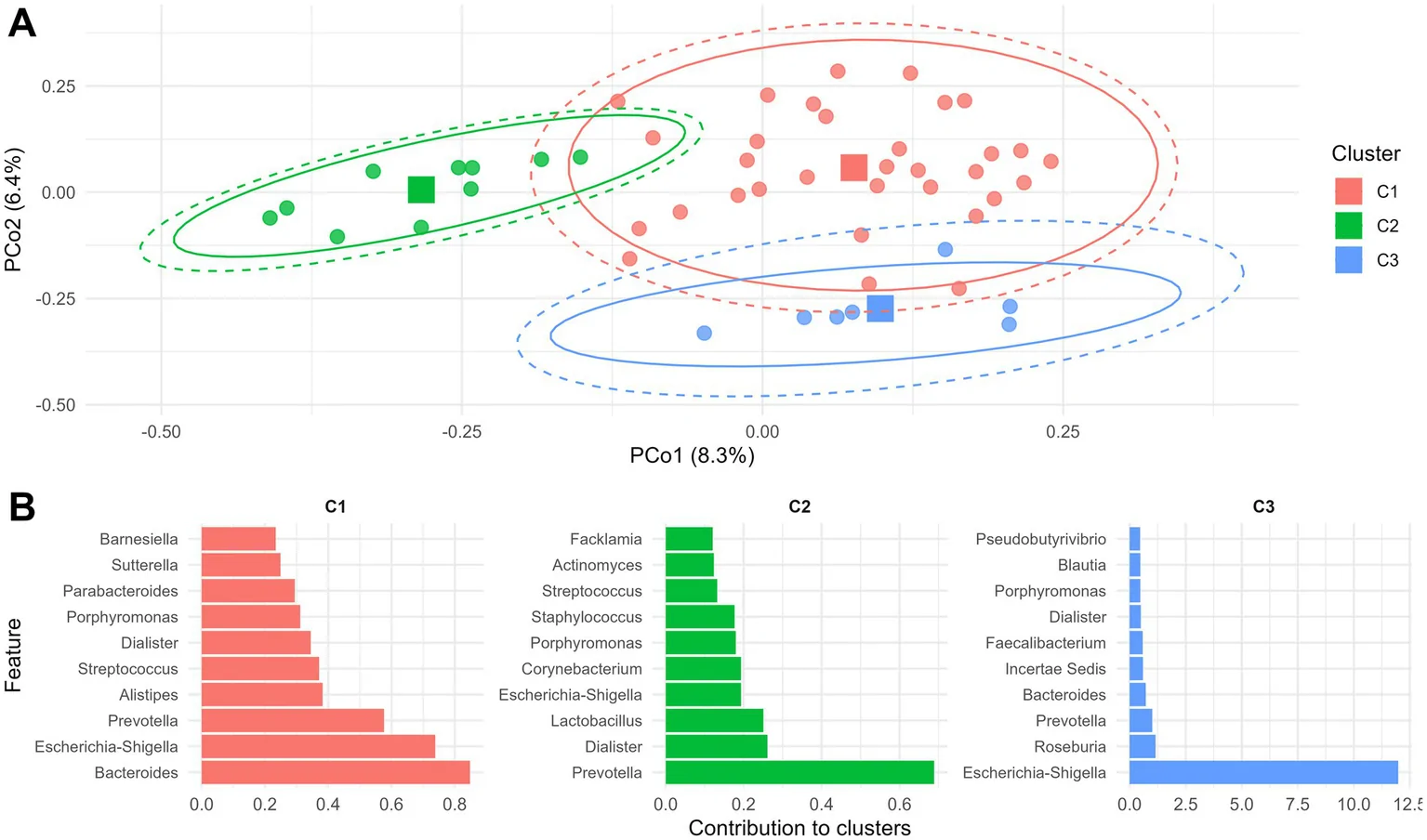
Gut microbiome community typing in EM patients. DMM modelling stratified patients into three distinct microbial community types (C1, C2, C3). **(A)** Principal coordinate analysis (PCoA) based on Canberra distances illustrates the clustering of samples into the three community types, with 95% confidence ellipses highlighting group separation. **(B)** Bar plots display the dominant genera characterizing each cluster.

Alpha diversity analyses across community types revealed that C2 displayed significantly lower Shannon diversity compared to C1 (Estimate = −0.50, Bonferroni-adjusted *p* = 0.0498) (Table 3). This finding suggests that individuals classified within C2 may harbor more uneven microbiome communities, potentially dominated by fewer taxa, which could reflect a dysbiotic state.

**Table 3:** Pairwise comparisons of alpha diversity indices (Shannon, Simpson, and richness) across community types (C1, C2, and C3).

Tested clusters	Diversity index	Estimate (*β*)	Standard Errors	95% CI	*P*-value	*P*-adj (Bonferroni)
C2 vs. C1	Shannon	−0.50304	0.236	[−0.966, −0.040]	0.0166	0.0498
C3 vs. C1	Shannon	−0.10525	0.274	[−0.641, 0.431]	0.35	1
C2 vs. C3	Shannon	−0.39779	0.327	[−1.039, 0.2434]	0.112	0.336
C2 vs. C1	Simpson	0.111359	0.067	[−0.020, 0.243]	0.0484	0.1452
C3 vs. C1	Simpson	0.058797	0.078	[−0.093, 0.211]	0.224	0.672
C2 vs. C3	Simpson	0.052562	0.093	[−0.129, 0.235]	0.286	0.858
C2 vs. C1	richness	−6.08851	10.524	[−26.716, 14.539]	0.281	0.843
C3 vs. C1	richness	15.34599	13.563	[−11.236, 41.928]	0.129	0.387
C2 vs. C3	richness	−21.4345	15.774	[−52.350, 9.481]	0.0871	0.2613

Estimates, standard errors, confidence intervals (95% CI), and *p*-values are reported. Multiple testing correction was applied using Bonferroni adjustment.

Beta diversity analyses (PERMANOVA) further underscored the distinctiveness of the clusters. Specifically, C1 differed significantly from both C2 (P-adjusted (P-adj) = 0.0015, R^2^ = 0.07) and C3 (P-adj = 0.0003, R^2^ = 0.06), indicating that these groups represent compositionally distinct microbial communities, albeit with modest effect sizes. By contrast, no significant differences were observed between C2 and C3 (P-adj = 0.7134), suggesting that these two clusters may share overlapping ecological configurations or reflect related dysbiotic states (Table 4). Concerning the beta dispersion evaluation, permutation test results are significant after Bonferroni correction when comparing C1 to C2 (Table 4). This suggests a higher variance within one of the tested groups. However, as shown in Figure 3A, the two clusters are well differentiated. Therefore, the beta diversity differences when considering these two groups can be caused by both the dispersion and microbiome composition of individuals belonging to the two clusters.

**Table 4:** Pairwise PERMANOVA (Adonis) and beta dispersion results comparing gut microbiome community structure between clusters (C1, C2, and C3).

Test	Sum of squares	R2	F-statistics	*P*-value	P-adj (Bonferroni)	P-adj_betadisper_ (Bonferroni)
C2 vs. C3	1.14	0.16	3.24	0.2378	0.7134	0.1155
C2 vs. C1	1.23	0.07	3.38	0.0005	0.0015	0.0084
C3 vs. C1	0.89	0.06	2.61	0.0001	0.0003	1.0000

Reported values include sum of squares, R^2^, F-statistics, raw *p*-values, and Bonferroni-adjusted *p*-values and Bonferroni-adjusted pairwise permutation tests *p*-values for beta dispersion.

### 3.4 Alpha diversity and EM-symptoms

Alpha diversity indices, including richness, Shannon, and Simpson, were evaluated in association with symptoms presence and severity (Mild vs. Intense pain, coded as a dichotomous variable) in the EM patients cohort (Supplementary Table 3).

A statistically significant association was observed for premenstrual pain severity. In details, premenstrual pain severity was associated with a higher Shannon (*β* = 0.277) and a lower Simpson (*β* = −0.74) indices (Table 5 and Figure 4). No significant differences were observed for species richness, indicating that both groups harbor a similar number of microbial taxa.

**Table 5:** Alpha diversity and EM premenstrual pain severity.

EM-symptom	Diversity	N	Estimate (*β*)	*P*-value	*P*-adj (Bonferroni)
Premenstrual pain (Mild vs. Intense pain)	Simpson	18(11)	0.277	4.00 × 10^−7^	3.359 × 10^−5^
	Shannon	18(11)	−0.74	1.99 × 10^−5^	1.674 × 10^−3^

The table reports the significative results identified between alpha diversity indices, and the EM-symptoms presence and severity (Mild vs. Intense pain, coded as a dichotomous variable). Reported statistics include number of individuals tested and number of individuals with intense pain (N), effect size [Estimate (*β*)], raw *p*-values (*P*-value), and *p*-values corrected using Bonferroni method [P-adj (Bonferroni)].

**Figure 4:**
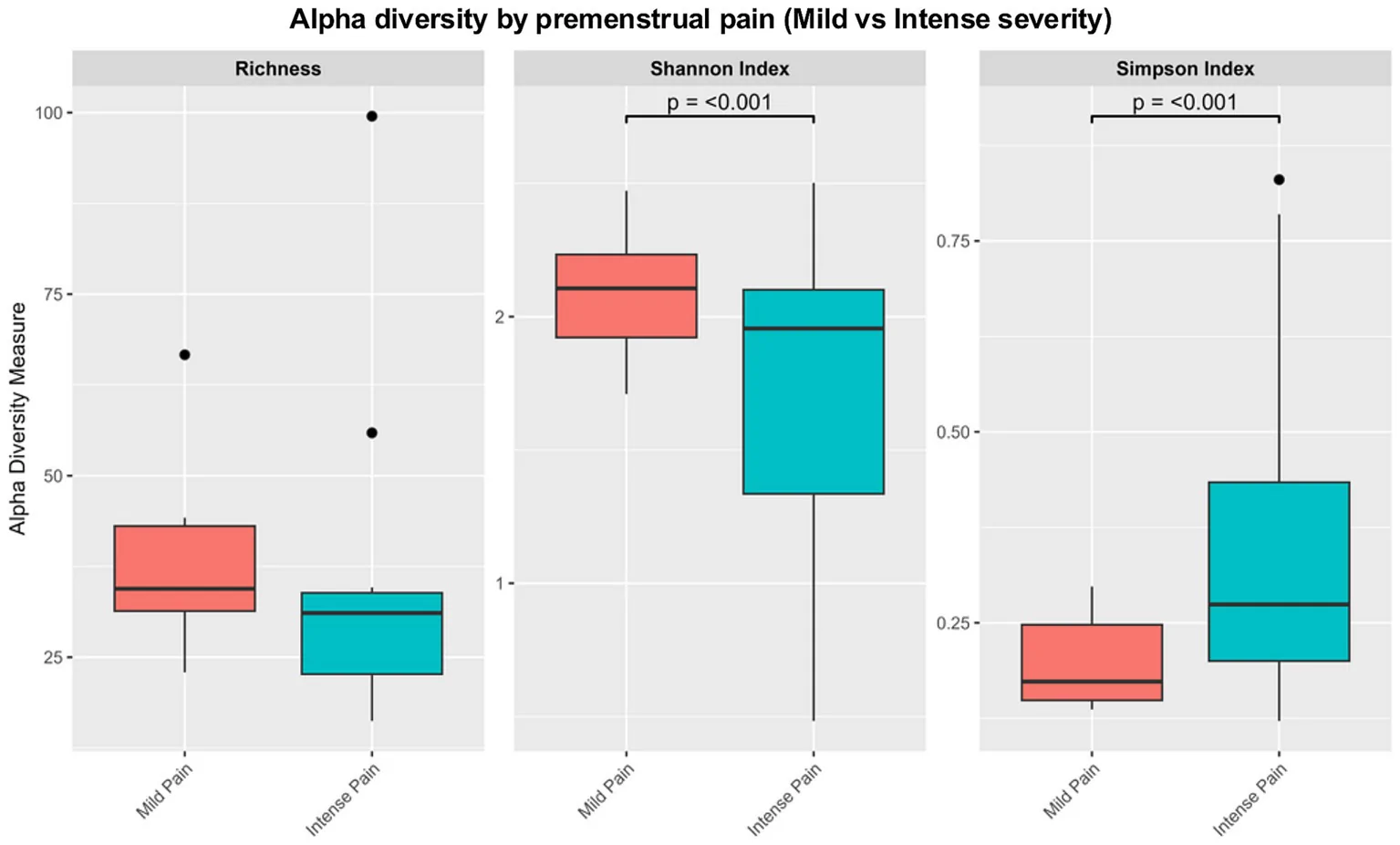
Alpha diversity and premenstrual pain severity. The figure reports boxplots showing the differences in alpha diversity metrics between two groups of EM patients classified by premenstrual pain severity. Mild Pain (NRS < 5) is indicated as dark-orange boxes and intense pain (NRS ≥ 5) in light-blue. The three panels display different alpha diversity measures: richness (number of species), Shannon Index and Simpson Index. Statistical analysis revealed a significant difference in the Shannon and Simpson indices between the two groups (*p*-value < 0.001 for both), indicating that the intense pain group has a higher microbial diversity compared to the mild pain group. No significant difference was observed for the richness metric.

Taken together, these results suggest that premenstrual pain severity may not be influenced by the number of microbial species but rather by differences in community structure, with greater evenness and reduced species dominance in patients with more severe pain, as reflected by Shannon and Simpson diversity indices.

### 3.5 Beta diversity and EM-features

Beta diversity analyses based on Canberra distances revealed significant associations between gut microbial community structure and EM features (Supplementary Table 4). Among EM-related traits, dysmenorrhea showed an association with microbiome composition (R^2^ = 0.13, P-adj = 0.0084), supporting the hypothesis that microbial shifts may contribute to pain severity in EM. Notably, EM stage (Early stage vs. Late stage) was also significantly associated with beta diversity (R^2^ = 0.12, P-adj = 0.0462), suggesting that microbiome shifts may accompany disease progression (Table 6). Furthermore, to verify if detected differences in beta diversity are caused by high dispersion within groups, rather than effective diversity differences between groups, the beta dispersion for significant EM features was evaluated. As shown in Table 6, EM stage showed no significant differences in beta dispersion after permutation test (*p*-value = 0.56). Conversely, dysmenorrhea showed significant differences after permutation test (*p*-value = <0.001), suggesting that the differences in beta diversity can be caused both by dispersion and individual gut microbiome composition, since the centroids of the two groups are well differentiated (Supplementary Figure 2).

**Table 6:** Beta diversity analyses based on Canberra distances testing associations between gut microbial community structure and EM-related features.

EM-features	P-value	R2	*F*-value	P-adj (Bonferroni)	P-value_betadisper_
Dysmenorrhea (presence/absence)	4.00×10^−4^	0.13	1.31	0.0084	1.00×10^−4^
EM stage (Early vs. Late)	0.0022	0.124	1.24	0.0462	0.56

Reported values include *P*-values, proportion of variance explained (R^2^), *F*-values, and Bonferroni-adjusted *P*-values [*P*-adj (Bonferroni)] and permutation test *P*-value for beta dispersion (*P*-value_betadisper_).

### 3.6 TGs-SNPs and alpha diversity

A list consisting of 12 extra-oral TG-SNPs with a functional role in the female reproductive system has been selected. Genotypes distribution according to each variant is displayed in Supplementary Table 5.

To evaluate potential associations between TG-SNPs and gut microbiome composition, microbiome diversity analyses were performed (Supplementary Table 6).

Regarding the alpha diversity, significant associations were detected in relation to four TGs variants (Table 7 and Figure 5). In particular, rs765007 (*TAS2R3* gene) showed a negative association with Simpson (*β* = −0.176) and a positive association with Shannon diversity (*β* = 0.454). A similar pattern was observed for *TAS2R4* and *TAS2R5* variants, namely rs2234001 and rs2234012, were consistently associated, respectively, with a lower Simpson (*β* = −0.185), and higher Shannon indices (*β* = 0.473) Finally, rs2708335 (*TAS2R14*) carriers display a higher Shannon diversity (*β* = 0.344). All together, these findings suggest that while the overall richness and evenness of microbial communities are increased, the dominance of specific taxa is reduced. These results suggest that genetic variation in TGs may influence microbial community structure by modulating host–microbe interactions, nutrient sensing, and mucosal immunity. The divergent effects observed on Shannon versus Simpson indices indicate that these variants may differentially affect dominant versus rare taxa.

**Table 7:** TG-SNPs and alpha diversity indices.

TG-SNPs	Diversity	Estimate (*β*)	*P*-value	P-adj (Bonferroni)
rs765007 (*TAS2R3*)	Simpson	−0.176	8.77 × 10^−11^	7.365 × 10^−9^
	Shannon	0.454	3.60 × 10^−8^	3.022 × 10^−6^
rs2234001 (*TAS2R4*)	Simpson	−0.185	9.49 × 10^−12^	7.972 × 10^−10^
	Shannon	0.473	3.21 × 10^−6^	2.697 × 10^−4^
rs2234012 (*TAS2R5*)	Simpson	−0.185	9.49 × 10^−12^	7.972 × 10^−10^
	Shannon	0.473	3.21 × 10^−6^	2.697 × 10^−4^
rs2708335 (*TAS2R14*)	Shannon	0.344	1.98 × 10^−4^	1.664 × 10^−2^

The table reports the significative results identified between TG-SNPs and alpha diversity indices. Reported statistics include effect size [Estimate (*β*)], raw *p*-values (*P*-value), and *p*-values corrected using Bonferroni method [*P*-adj (Bonferroni)].

**Figure 5:**
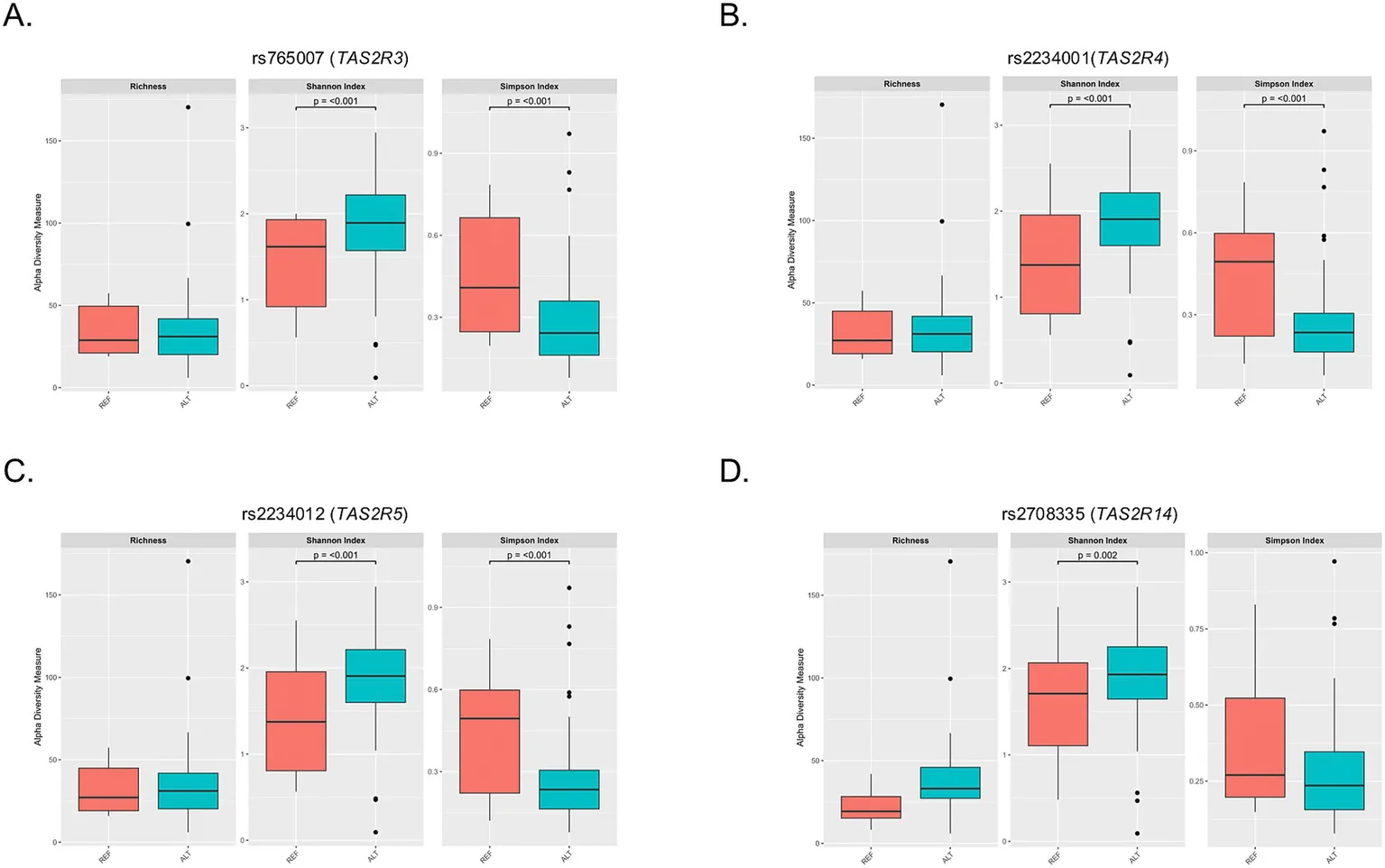
TG-SNPs and alpha diversity. The figure reports boxplots showing the alpha diversity metrics according to TG-SNPs genotype carriers. The three panels display different alpha diversity measures: richness (number of species), Shannon Index and Simpson Index. Statistical analysis revealed a significant difference in the Shannon and Simpson indices between the two groups, with the exception of panel D (*p*-value < 0.001 for both). No significant difference was observed for the richness metric.

### 3.7 TGs-SNPs and beta diversity

Beta diversity was assessed in relation to TGs-SNPs genotype status (Supplementary Table 7). Nine SNPs within the *TAS2R14* gene (namely, rs1901188, rs2900582, rs10845262, rs7306372, rs2708335, rs4763232, rs7313683, rs7488102, and rs3851584) exhibited consistent and significant associations with beta diversity [P-adj (Bonferroni) < 0.05) (Table 8].

**Table 8:** Results of beta diversity analysis according to TGs genetic variants.

TGs-SNPs	*P*-value	R2	*F*-value	P-adj (Bonferroni)
rs10845262 (*TAS2R14*)	5.00 × 10^−4^	0.128	1.29	0.0105
rs2708335 (*TAS2R14*)	7.00 × 10^−4^	0.127	1.28	0.0147
rs3851584 (*TAS2R14*)	0.002	0.123	1.23	0.0462
rs4763232 (*TAS2R14*)	8.00 × 10^−4^	0.125	1.26	0.0168
rs7306372 (*TAS2R14*)	6.00 × 10^−4^	0.128	1.29	0.0126
rs7313683 (*TAS2R14*)	0.0016	0.126	1.26	0.0336
rs7488102 (*TAS2R14*)	0.0017	0.124	1.25	0.0357
rs1901188 (*TAS2R14*)	4.00 × 10^−4^	0.127	1.28	0.0084
rs2900582 (*TAS2R14*)	4.00 × 10^−4^	0.128	1.29	0.0084

Statistically significant differences in microbial community composition were observed for the TG-SNPs shown below. Reported statistics include raw *p*-values (*P*-value), R^2^ (variance explained), *F*-values, and *p*-values corrected using Bonferroni method.

These findings could suggest that genetic variation in *TAS2R14* may thus influence microbiome composition through altered chemosensory signaling, impacting epithelial barrier responses and shaping host tolerance to specific bacterial taxa. Given the established link between the *TAS2R14* gene and fertility outcomes, these results could support the hypothesis that this extra-oral TG may be associated with the gut-reproductive tract axis, potentially contributing to the pathophysiology of EM and fertility issues.

### 3.8 Gut microbiota composition influences EM features

Linear regression models were conducted to identify potential associations between gut microbial taxa and the severity of EM-related features, and TGs-SNPs genotypes (Supplementary Table 8).

Table 9 reports the detected statistically significant associations; in detail, a lower abundance of taxa within the order *Lachnospirales*, including the families *Lachnospiraceae* and *Ruminococcaceae*, and several representative genera (*Blautia*, *Faecalibacterium*, *Roseburia*, *Pseudobutyrivibrio*, and *Lachnospiraceae_ND3007_group*), were associated with the presence of dysmenorrhea. Considering that these bacterial groups are SCFA-producers and widely recognized for their anti-inflammatory roles (Zhang et al., 2018), their reduced abundance in EM patients experiencing dysmenorrhea suggests a potential disruption of butyrate-mediated anti-inflammatory pathways, which may exacerbate inflammatory responses and symptom severity.

**Table 9:** Linear regression results showing associations between gut microbiota taxa (at the phylum, class, order, family, and genus levels), and EM-related phenotypes.

Bacteria	Rank	EM’s related phenotypes	N_individuals	Estimate (*β*)	Standard Error	*P*-value	95% CI	P-adj Bonferroni
*Lachnospirales*	Order	Dysmenorrhea (presence/absence)	50	−0.24	0.063	0.000426	[−0.368,-0.113]	0.014
*Lachnospiraceae*	Family			−0.207	0.054	0.000375	[−0.316,-0.099]	0.012
*Ruminococcaceae*	Family			−0.281	0.08	0.001072	[−0.44286,-0.11933]	0.035
*Blautia*	Genus			−0.381	0.096	0.000268	[−0.574,-0.187]	0.009
*Faecalibacterium*	Genus			−0.316	0.07	4.83E-05	[−0.457,-0.174]	0.001
*Lachnospiraceae.ND3007.group*	Genus			−0.656	0.161	0.000195	[−0.982,-0.331]	0.006
*Pseudobutyrivibrio*	Genus			−0.356	0.102	0.001096	[−0.561,-0.151]	0.036
*Roseburia*	Genus			−0.23	0.056	0.000184	[−0.344,-0.117]	0.006
*Prevotella_9*	Genus	Dysuria (presence/absence)	49	0.136	0.037	0.000702	[0.061,0.212]	0.023
*Mobiluncus*	Genus	Infertility (presence/absence)	50	0.327	0.092	0.000875	[0.142,0.511]	0.029

Linear regression models (Model: $\mathrm{Tax}a_{\mathrm{CLR}-\text{transformed}}∼EM_{\text{related phenotype}}+\text{Covariates}$ ) were performed at different taxonomic levels (order, family, genus) using relative abundances of bacterial taxa as the outcome. Reported values include number of individuals with complete observation tested (N_individuals), effect estimates (*β*), standard errors, 95% confidence intervals (95% CI), and *p*-values corrected for multiple testing using Bonferroni methods.

Conversely, an increased abundance of *Prevotella_9* was positively associated with dysuria, highlighting a potential role of this genus in lower urinary tract-related symptoms in EM. Similarly, *Mobiluncus*, a genus associated with reproductive tract dysbiosis (Hasegawa et al., 2025), was positively associated with infertility in EM patients. This finding may support the hypothesis of a functional gut-reproductive tract axis, whereby specific microbial signatures could impair reproductive potential through systemic immune modulation or direct effects on the microbiome of the reproductive tract.

No statistically significant associations were detected between gut microbial taxa and TG-SNPs (Supplementary Table 9).

### 3.9 EM symptoms influence gut microbiota composition

Linear regression models were conducted to identify potential associations between the severity of EM-related symptoms, TG-SNPs and gut microbial taxa (Supplementary Table 10).

In details, an increased premenstrual pain severity was associated with a reduced relative abundance of taxa within the order *Burkholderiales* (*β* = −2.265) and the family *Sutterellaceae* (*β* = −1.563). Similarly, higher postmenstrual pain severity was associated with a lower abundance of *Bifidobacteriales* (*β* = −4.932) and *Bifidobacteriaceae* (*β* = −4.134). At the genus level, a higher dyschezia severity was negatively associated with the abundance of *Bifidobacterium* (*β* = −2.005) (Table 10).

**Table 10:** Linear regression results showing associations between EM symptom severity and gut microbiota taxa.

EM-symptom	N_individuals	Bacteria	Rank	Estimate (*β*)	Standard Error	95% CI	P-adj (Bonferroni)
Premenstrual pain	18	*Burkholderiales*	Order	−2.265	0.560	[−3.484, −1.046]	0.011
Premenstrual pain	18	*Sutterellaceae*	Family	−1.563	0.430	[−2.499, −0.626]	0.023
Post-menstrual pain	11	*Bifidobacteriales*	Order	−4.932	1.071	[−7.686, −2.177]	0.041
Post-menstrual pain	11	*Bifidobacteriaceae*	Family	−4.134	0.912	[−6.478, −1.790]	0.043
Dyschezia	14	*Bifidobacterium*	Genus	−2.005	0.554	[−3.284, −0.727]	0.048

Linear regression models (Model: $\mathrm{EM}-\text{symptoms intensity}∼\mathrm{Tax}a_{\mathrm{CLR}-\text{transformed}}+\text{Covariate}$ s) were performed to evaluate association between EM symptom severity and different taxonomic levels (order, family, genus) using symptom severity as the outcome. Reported values include number of individuals with complete observation tested (N_individuals), effect estimates (*β*), standard errors, 95% confidence intervals (95% CI), and *p*-values corrected for multiple testing using Bonferroni method.

These results suggest that increased premenstrual severity in EM could be linked to the depletion of specific bacterial groups, particularly within the *Bifidobacterium* lineage, which may play a role in pain modulation and gut–reproductive system interaction. No statistically significant associations were detected between gut microbial taxa and TG-SNPs.

## 4 Discussion

EM is a common and multifactorial disorder, where genetic and environmental factors determine disease onset and progression. Despite decades of research, the causal mechanisms connecting the ectopic endometrial lesions onset to the wide spectrum of symptoms experienced by affected women remain only partially understood.

Increasing attention has been directed toward the role of the gut microbiome, which is now recognized as a central modulator of systemic inflammation, immune responses, and even reproductive function (Baușic et al., 2025). Alterations in microbiome community composition have been consistently reported in EM, suggesting that dysbiosis may not only contribute to the inflammatory environment that may sustain lesions growth, but also to the severity of EM.

Specifically, this microbiome-driven inflammatory cascade is increasingly recognized as a key pathophysiological feature across a broad spectrum of endometrial dysfunctions. Recent evidence highlights that gynecological microbiota dysbiosis, through the epithelial/mucosal barrier disruption reshape also local tissue microenvironment, contributing to localized genetic instability and pathological progression in both benign and malignant gynecological conditions, including endometrial cancer (Aquino et al., 2024).

Within this framework, the present study aimed to investigate the relationship between gut microbiome composition and EM symptom severity in a well-characterized cohort of 50 women with surgically confirmed disease. By integrating clinical data with microbial diversity and taxonomic analyses, we sought to elucidate whether specific microbiome signatures may underlie symptom burden and EM-related features, independent of classical measures of disease extent such as rASRM stage.

Among the spectrum of EM-related symptoms collected in our study, dysmenorrhea, ovulation pain, and dyspareunia were the most frequent in our cohort, reported, respectively, by the 66, 47 and 46% of the patients. These findings underscore the high clinical burden associated with EM, consistent with prior studies reporting substantial quality-of-life impairment among affected women (Da Broi et al., 2019b; Horton et al., 2019). Furthermore, our results corroborate the absence of correlation between anatomical disease severity and symptom intensity. Indeed, no significant associations were observed between rASRM stage and pain severity, reinforcing the evidence that disease stage alone does not fully capture the symptomatic burden of EM. This finding has relevant clinical implications, highlighting the need for symptom-driven management rather than stage-driven treatment strategies.

In this context, we further explored potential relationships between EM symptoms and gut microbiome composition, given that the microbiota has been increasingly recognized as a potential contributor to EM severity (Baușic et al., 2025).

In our cohort, the gut microbiome was characterized by a predominance of the genera *Escherichia-Shigella*, *Prevotella*, and *Lactobacillus*, with the phyla *Bacteroidota*, *Firmicutes*, and *Proteobacteria* being most represented. Notably, the increased abundance of *Escherichia-Shigella* and *Bacteroidota* observed in our cohort is consistent with previous studies reporting microbiome dysbiosis in EM patients (Khan et al., 2018). These alterations of the gut microbiota may have pathophysiological relevance, as both *Escherichia–Shigella* and members of *Proteobacteria* are well-known contributors to epithelial barrier disruption, thus promoting systemic immune activation and chronic inflammation, which are hallmarks of EM progression. Interestingly, no differences in microbiome composition were observed between early and late rASRM stages. Currently, at the best of our knowledge, only one study compared early- versus late-stage EM in microbiome analyses, reporting no differences in alpha diversity but distinct variations in community structure (Cai et al., 2025). Taken together, these findings suggest that the variations in microbial composition among EM patients may not be strictly dependent on disease stage, thus highlighting the need to move beyond classical staging systems when evaluating the role of the microbiome in EM. Moreover, integrating microbial profiling with patient-reported symptom severity to gain could provide a deeper understanding of host–microbe interactions in this condition.

To gain further insights, an enterotype analysis was employed to define gut microbiome community types. Three distinct enterotypes have been defined: C1, predominantly enriched in *Bacteroides*; C2, characterized by a dominance of *Prevotella*; and C3, notably distinguished by a high abundance of *Escherichia–Shigella.* From a clinical perspective, these three community types may carry important implications for EM pathophysiology and symptoms variability. Indeed, according to the literature, although the composition of the gut microbiome varies greatly between individuals, previous studies defined three main enterotypes: the *Bacteroides*-dominant, *Prevotella*-dominant, and *Ruminococcus*-dominant ones (Arumugam et al., 2011). Hence, in our study, the C3 enterotype, marked by *Escherichia–Shigella*, represents a shift from the classical enterotypes, suggesting that disease-specific factors may influence microbial community composition. From a clinical perspective, this enterotype may indicate a dysbiotic state linked to intestinal inflammation or barrier dysfunction, potentially involved in exacerbating disease severity or symptom variability. Notably, recent reports suggested that *a Escherichia–Shigella* enrichment in the gut could be linked to gynecological disorders and infertility (Baușic et al., 2025), since it could contribute to systemic immune activation, fostering a pro-inflammatory state that may worsen EM-associated symptoms or even promote lesion persistence.

Furthermore, it was investigated whether the microbiome composition may influence EM-symptoms. Previous studies reported that women presenting with premenstrual syndrome have a decreased beta diversity in comparison with healthy volunteers (Takeda et al., 2022); here, for the first time, we observed significant associations between alpha diversity metrics and premenstrual pain severity. Patients reporting intense premenstrual pain displayed higher Shannon and lower Simpson indices compared to those with mild pain, despite no difference in species richness. Hence, results indicate that gut microbiome dysregulation may play a key role in shaping premenstrual pain perception in EM.

Regarding the beta diversity analysis, dysmenorrhea and EM stage (Early vs. Late stages) resulted significant from the PERMANOVA test. Although the effect sizes were modest, this study provides the first evidence of beta diversity differences between EM stages. These preliminary findings are promising and warrant confirmation in larger cohorts. Overall, the results reinforce the notion that alterations in gut microbial composition may contribute to symptom manifestation and disease severity in EM. Consequently, gut microbial profiling holds potential as a strategy for patient stratification and the development of personalized management approaches.

Starting from these considerations, given the well-established role of the gut microbiota in modulating systemic inflammation and nociceptive pathways, it could be relevant to identify specific bacterial taxa that drive the observed associations, ultimately paving the way toward the development of microbiome-targeted strategies.

In this line, linear regression models were conducted to detect possible associations between specific microbial taxa and the severity of EM-symptoms.

In detail, a lower abundance of taxa within the order *Lachnospirales*, including the families *Lachnospiraceae* and *Ruminococcaceae* were associated with the presence of dysmenorrhea. These bacterial groups are among the major butyrate producers in the gut microbiome and are widely recognized for their anti-inflammatory role (Zhang et al., 2018). Their reduced abundance in EM patients experiencing dysmenorrhea suggests a potential disruption of butyrate-mediated immunoregulatory pathways, which may exacerbate inflammatory responses and pain perception. This finding aligns with prior evidence linking diminished butyrate-producing bacteria to other chronic pain and inflammatory disorders (Parada-Venegas et al., 2025). Conversely, an increased abundance of *Prevotella_9* was positively associated with dysuria, suggesting a potential role of this genus in lower urinary tract symptomatology in EM. Members of *Prevotella* have been implicated in mucosal inflammation and epithelial barrier disruption (Larsen, 2017a), and their enrichment may therefore contribute to exacerbation of urogenital symptoms. Similarly, *Mobiluncus*, a genus associated with reproductive tract dysbiosis (Hasegawa et al., 2025), was positively associated with infertility in EM patients. This supports the hypothesis of a functional gut–reproductive tract axis, whereby specific microbial signatures could impair reproductive potential through systemic immune modulation or direct effects on the genital tract microbiome.

Taken together, these associations suggest that reduced representation of beneficial, butyrate-producing taxa may predispose to heightened pelvic pain, while enrichment of potentially pathogenic or pro-inflammatory genera such as *Prevotella* and *Mobiluncus* may exacerbate urinary and fertility-related symptoms. Although causality cannot be established, these findings strengthen the rationale for targeting microbial communities—through dietary interventions, probiotics, or microbiota-directed therapies—as potential avenues for symptom alleviation and fertility improvement in EM.

In more detail, linear regression models detected associations between increased dysmenorrhea severity and the reduction of SCFA-producing taxa, including *Faecalibacterium* (*β* = −0.316), *Roseburia* (*β* = −0.231) (Duncan et al., 2002; Louis and Flint, 2017), *Blautia* (*β* = −0.381), (Holmberg et al., 2024), and members of the *Lachnospiraceae* family (Zaplana et al., 2024). Butyrate is an SCFA known for its anti-inflammatory and epithelial barrier-supporting functions; its depletion could exacerbate local inflammation and visceral hypersensitivity, contributing to dysmenorrhea (Siddiqui and Cresci, 2021). Conversely, dysuria severity was positively associated with *Prevotella 9*, a genus often linked to pro-inflammatory states and mucosal dysbiosis (Larsen, 2017b), further supporting the hypothesis of the existence of symptom-specific microbial signatures in EM.

Beyond microbiome–symptom associations, we further explored the contribution of host genetic variation, with a specific focus on the unexplored role of TGs. These receptors, increasingly recognized as key modulators of host–microbe crosstalk and immune–metabolism signaling, may represent a novel and largely unknown field of interest in the context of EM.

In our study, significant associations between *TAS2R3, TAS2R4, TAS2R5,* and *TAS2R14* SNPs and alpha diversity indices were observed. This is consistent with prior evidence indicating that TAS2Rs expressed in the gastrointestinal tract act as nutrient and chemical sensors, modulating hormone secretion, immune responses, and microbial community dynamics (Lee and Cohen, 2015; Feng et al., 2018b). Given that TAS2Rs are directly involved in the perception of bitter compounds from dietary sources, interindividual genetic variability in these receptors may influence food preferences and dietary patterns (Li and Zhang, 2014), which in turn modulate gut microbiome composition. Dysregulation of these signaling pathways could therefore impact diet–gene–microbiome interactions, thus contributing to dysbiosis and pro-inflammatory states, which are highly relevant to EM onset and progression.

Moreover, nine *TAS2R14* SNPs were found to be associated with gut microbial *β*-diversity. Notably, *TAS2R14* encodes a bitter taste receptor expressed in the gastrointestinal tract and female reproductive system, contributing to nutrient sensing, innate immune responses, and microbial surveillance (Semplici et al., 2021). Variations in this receptor may alter epithelial and immune signaling, shaping the gut microbial ecosystem and, consequently, influencing reproductive health. Thus, genetic variation in TAS2Rs may simultaneously influence the gut microbial balance and ovarian function, acting as a molecular bridge between microbiome, and reproductive health.

Taken together, these findings support a novel role for *TAS2Rs* genetic variation in EM pathophysiology, not only through shaping the intestinal microbial community but also by potentially modulating fertility-related processes. This dual involvement highlights *TAS2Rs* as promising candidates for future mechanistic studies aimed at clarifying their contribution to the gut-reproductive tract axis.

Further studies in larger cohorts will be needed to confirm the detected relationships and to better elucidate the mechanistic links between microbiome alterations, EM pathophysiology, and *TAS2R* genetics. Additionally, replication of the findings in larger cohorts will be essential to validate host genetic influences on gut microbiome composition and EM manifestations.

The strength of this work lies in the deep clinical phenotyping of the cohort with both gut microbiome profiling and *TAS2Rs,* which offers a novel framework for understanding the host-microbe axis within this pathology. Conversely, certain limitations must be acknowledged. First, the modest sample size modest inherently restricts our statistical power to identify subtler gene-microbiome interactions. Second, this is an exploratory, case-only study; the absence of an unaffected, healthy control cohort limits our ability to generalize these microbiome configurations as definitive diagnostic biomarkers. Third, due to the urgent nature of the surgical interventions for EM in our clinical setting, it was not possible to standardize sample collection according to a specific phase of the menstrual cycle, which remains a potential unmeasured confounder. Finally, while 16S sequencing is a reliable and well-established tool for characterizing community taxonomic composition, it offers limited resolution at the species level and does not capture functional genomic content. Future larger-scale, multi-centre investigations utilizing shotgun metagenomics are warranted, as high-throughput shotgun sequencing offers broader investigative perspectives by directly mapping metabolic and functional pathways alongside higher-resolution taxonomic profiling. Finally, considering that *TAS2R* variants heavily influence dietary compliance, longitudinal food frequency questionnaire data will be relevant to disentangle the relative contributions of host genetics and dietary habits in shaping the gut microbiota structure, thereby uncovering complex gene-diet-microbiota interactions in EM pathophysiology.

Overall, our study underscores the multifactorial nature of EM, highlighting how both gut microbiota and genetic factors play a critical role in modulating symptom severity. Our findings contribute to the emerging evidence of a bidirectional cross-talk between microbiome and EM, expanding current knowledge on the potential mechanisms that contribute to EM pathophysiology.

In conclusion, these findings support the need for a personalized approach for EM management. Indeed, future identification of microbiome-derived biomarkers may facilitate patients’ stratification, allowing clinicians to tailor management strategies for symptom severity. Furthermore, a deeper understanding of host–microbiome interactions may open new therapeutic avenues, such as personalized dietary interventions, prebiotic or probiotic supplementation, or microbiota-targeted pharmacological strategies aimed at mitigating inflammation, alleviating pain, and potentially improving fertility outcomes. Ultimately, advancing this line of research could contribute to more precise, mechanism-based, and patient-centered care for women with EM.

## Data Availability

The raw data supporting the conclusions of this article are not publicly available to protect participant confidentiality and privacy. Requests to access the datasets should be directed to the corresponding author.
